# A 7-year longitudinal model of math and receptive vocabulary in primary school

**DOI:** 10.3389/fpsyg.2026.1689488

**Published:** 2026-05-01

**Authors:** Carolina Álvarez, Dénes Szücs

**Affiliations:** Department of Psychology, Centre for Neuroscience in Education, University of Cambridge, Cambridge, United Kingdom

**Keywords:** academic achievement, executive functions, math, preschool, receptive vocabulary

## Abstract

**Introduction:**

Identifying early predictors of vocabulary and math skills can support children’s academic development. Here, we investigated indirect longitudinal paths promoting children’s math and receptive vocabulary. We aimed to understand how mother’s socio-economic status and cognitive skills and children’s language ability measured when children were of 3 years of age (T1) are connected to age 11 (T3) math and receptive vocabulary outcome measures through the mediating effect of the availability of home learning materials, children’s executive functions and receptive vocabulary measured at age 5 (T2).

**Methods:**

We used structural equation modelling (SEM) to test our mediation model in a sample of 3,757 Chilean children (1,858 girls, 1,899 boys).

**Results:**

We found that the strongest indirect path started with early language skills predicting children’s executive functions in preschool, which in turn promoted both math and vocabulary in primary school.

**Discussion:**

We discuss the theoretical and educational implications of our findings.

## 1 Introduction

Early math and vocabulary skills provide the foundation for more advanced academic content, such as arithmetic and algebra (Leyva et al., 2021) and reading comprehension (Silva and Cain, 2015), respectively. Children’s understanding of math operations, their ability to fluently execute procedures, and their knowledge of how to apply these operations to problem solving can impact their math learning in the following school years. Likewise, children’s vocabulary knowledge supports their inferential and literal comprehension of a text. Moreover, there is evidence that math and vocabulary skills are interconnected (Duncan et al., 2007; Purpura et al., 2011). Given these relationships, identifying early predictors of these skills provide valuable opportunities for policymakers, teachers, and parents to support children before school entry, informing the design of timely interventions, guide the allocation of resources, and supporting the implementation of practices that foster early math and vocabulary skills. These efforts can help prevent later academic difficulties and promote educational equity.

This study is framed within Bronfenbrenner’s bioecological model of human development (Bronfenbrenner and Morris, 2007). This theory proposes that development occurs through dynamic proximal processes—interactions over time between the child and their environment. Environmental contexts are understood as nested systems that interact and influence development. The microsystem represents the immediate setting in which the developing child regularly interacts.

Within the microsystem, factors such as maternal cognitive skills, family socioeconomic status, and the availability of learning materials at home can significantly influence children’s early skills and development. Therefore, here we explore a comprehensive longitudinal model, including three potential mediators in the preschool years (home learning materials, children’s executive functions and receptive vocabulary) linking family socioeconomic status, maternal cognitive skills and children’s language in infancy with math and receptive vocabulary in middle childhood. We analyze this model using a nationally representative sample from the Early Childhood Longitudinal Survey (ELPI) from Chile, a population often underrepresented in research. The large sample allowed us to estimate precise effect sizes, contributing to the existing body of research by adding evidence from Latin America, a region with high educational inequalities.

### 1.1 International findings

There have been important efforts to test models predicting children’s math or vocabulary. Evidence from the NICHD (National Institute of Child Health and Human Development) Study of Early Child Care and Youth Development has highlighted the importance of 4-year-old children’s ability to inhibit irrelevant information and maintain attention on their task (also known as Executive Functions, EFs) as a mediator between parental education and math achievement and receptive vocabulary in first grade (Sektnan et al., 2010, *N* = 1,298; Waters et al., 2021, *N* = 1,273). Additionally, an enriched home environment was shown to buffer some of the negative effects of low maternal education on children’s math achievement in first grade (Zadeh et al., 2010, *N* = 1,093). Other US studies have also highlighted the role of the home environment for the development of children’s vocabulary (Froiland et al., 2013, *N* = 551; Gonzalez et al., 2017, *N* = 252). The Family Life Project (children born between 2003 and 2004 in the US) found that early poverty risk affected math and reading via EFs (Perry et al., 2018, *N* = 1,292); whereas children with higher receptive vocabulary and EFs in pre-K had higher levels of math and reading comprehension in primary school (Burchinal et al., 2020, *N* = 1,292). These models are informative on the role of EFs, the home environment, and receptive vocabulary as mediators between parental socioeconomic measures and children’s outcomes.

There have also been large studies in Australia (such as The Longitudinal Study of Australian Children; Shahaeian et al., 2018, *N* = 4,768), Singapore (Ng et al., 2021, *N* = 1,037), and Canada (Wade et al., 2018, *N* = 501), which have similarly found that maternal education and linguistic input in infancy can influence early math through the development of receptive vocabulary or EFs in the preschool years.

Although a range of previous studies have tested different models predicting children’s math and receptive vocabulary, many are now based on decades-old data, include a limited number of measures, have non-representative samples, focus exclusively on Western countries, and few used longitudinal models covering different stages of child development (Burchinal et al., 2020; Lombardi and Dearing, 2021; Wade et al., 2018; Waters et al., 2021). Our study aimed to address these limitations. We will expand on these models, covering 7 years across three time points, including previously tested predictors (socioeconomic status, SES) and mediators (children’s EFs and receptive vocabulary, and learning materials), novel predictors (mothers’ cognitive skills) and three math skills (calculation, fluency, and applied problems) to allow for a more comprehensive assessment and understanding of longitudinal paths predicting math and vocabulary.

### 1.2 Latin American and Chilean findings

High levels of poverty and inequality of resources in the Latin American region are severe risk factors for child development. For example, most Latin American children reach the end of kindergarten with lower vocabulary than children from developed countries (OECD, 2017; Strasser et al., 2016). Cross-national studies have found that parents with more than 12 years of education had 6–17-year-old children with higher receptive vocabulary (Olabarrieta-Landa et al., 2017, *N* = 4,373, nine Latin American countries and Spain); and gender differences such that boys had higher math achievement than girls in sixth grade (Liu et al., 2020, *N* = 57,476, 15 Latin American countries). These studies draw attention to the educational segregation in the region, with lower income students often attending underfunded public schools.

Chile has the highest level of inequality among OECD countries (OECD, 2016) and Latin-American countries (CEPAL, 2012). The Programme for International Student Assessment (PISA) and national standardized tests show large SES and gender achievement gaps in math, reading, and science (Montoya et al., 2019). Besides, Chile has 19% tax rate on books (one of the highest in the world), and therefore many low and middle SES families cannot afford them (Mendive et al., 2020).

More local evidence is needed to advise policy makers and help improve the design and evaluation of interventions and public policies focusing on childhood (Narea, 2016). To our knowledge, ours is the first study in Latin America exploring a longitudinal mediation model predicting math and receptive vocabulary in a large sample.

### 1.3 The present study

Building on the existent international and national literature, we designed a comprehensive model using a nationally representative sample of 3,757 children from Chile, enrolled in the Early Childhood Longitudinal Survey (ELPI) across three waves. Our main research question was: do home learning materials, children’s EFs and receptive vocabulary at age 5 (T2) mediate the relation between mother’s SES and cognitive skills and children’s language at age 3 (T1) with children’s math and receptive vocabulary at age 11 (T3)? We used structural equation modeling (SEM) to test this model. Our large sample size allows us to calculate precise effect sizes, providing useful evidence for stakeholders to support children’s math and vocabulary development. For completeness, we investigated whether this model applied to both girls and boys, as this would have implications for the generalizability of results.

First, we expected that mother’s SES and cognitive skills and children’s language at T1 would be related to home learning materials and children’s EFs and receptive vocabulary at T2 (Froiland et al., 2013; Wade et al., 2018).

Second, we expected that home learning materials and children’s EFs and receptive vocabulary at T2 would be related to children’s receptive vocabulary and math at T3 (Ng et al., 2021; Perry et al., 2018; Shahaeian et al., 2018).

## 2 Materials and methods

We analyzed longitudinal data from a nationally representative sample from ELPI (Encuesta Longitudinal de Primera Infancia; Early Childhood Longitudinal Survey), collected in Chile in three waves so far (2010, 2012, and 2017; Ministerio de Desarrollo Social y Familia, 2019). The ELPI sample is representative of children born between January 1, 2006, and August 31, 2009, based on birth records from the Chilean Civil Registry and Identification Service (Coddington et al., 2014). ELPI aims to assess child development and characterize the home environment and immediate context to inform public policy.

### 2.1 Ethics

Ethical evaluation of ELPI 2010 and 2012 was conducted by Universidad de Chile, whereas in 2017 it was conducted by Pontificia Universidad Católica de Chile. Databases are available online for research purposes (Ministerio de Desarrollo Social y Familia, 2019).

### 2.2 Participants

To test our proposed model, we analyzed a sub-sample of ELPI. The inclusion criteria were children aged between 30 and 58 months old in 2010 (T1) (as one of the T1 measures required children to be 30 months or older when tested) and having complete follow-up data on the selected measures from 2012 (T2) to 2017 (T3). Our final sample consisted of 3,757 children (1,858 girls, 1,899 boys; *M* age at T1 = 41.05 months; *SD* = 6.85; range = 30–58 months; *M* age at T2 = 66.94 months; *SD* = 6.93; range = 52–82 months; *M* age at T3 = 135.14 months; *SD* = 7.15; range = 117–151 months). A total of 3,578 children who met the age inclusion criteria at T1 did not have complete follow-up data on the selected measures at T2 and T3 and therefore were not included in this study. We did not find significant differences (*p* < 0.001) between the missing sample (without follow-up data) and the sample of this study. Please see Supplementary Tables 1, 2 for more detail.

The highest level of education of most main caregivers at T1 was high school (0.5% no formal education, 19.4% primary school; 67.9% high school; 6.4% technical studies, 5.1% college degree or higher), and they were between 16 and 71 years old (*M* age = 30.95 years; *SD* = 7.46). 98.5% of the main caregivers corresponded to the biological mother (0.2% biological father, 0.3% adoptive mother, and 1% other family members).

Family income (i.e., the average household income over the past 12 months, considering all sources of income) was reported by the main caregiver at T1 and ranged between $15,000–21,600,000 Chilean pesos (*M* = 458,354, *SD* = 898,325). At the national level, the first, second, third, fourth, and fifth quintile had 5, 9, 13, 20, and 53% of the total income in 2011, respectively (World Bank, 2023). In 2010, the income of the 90th percentile was $931,512, whereas the income of the 10th percentile was $71,655 (*M* = 430,540; INE, 2015). The Gini index, measuring inequality on a scale from 0 to 1 (higher values indicate higher inequality), was 0.42 in 2009 (CEPALSTAT, 2023).

### 2.3 Procedure

To construct the ELPI sample, the 82 largest Chilean municipalities and a regional capital were automatically included. The remaining 263 municipalities were grouped in clusters by geographic region and ordered by income per capita and number of children in the target age range. One municipality was randomly chosen from each of these 33 clusters. This amounted to a total of 116 municipalities. Eligible children were stratified by gender and age and randomly drawn from across these 116 municipalities (For more details please see Coddington et al., 2014).

For data collection, pairs of trained research assistants conducted two home visits to families. Written consent was provided by the main caregiver (in 2010, 2012, 2017) and the child (in 2017). Children could stop participating at any moment. During the first visit, the main caregiver completed a sociodemographic questionnaire. In the second visit, the child and caregiver were individually assessed. Both visits were completed usually within 2 weeks.

The current study draws on the sociodemographic questionnaire for information on maternal education, family income, and home learning materials. Mothers were assessed on cognitive skills (vocabulary and WM), and children were assessed on language, receptive vocabulary, EFs, and math skills. Table 1 details the measures included, the instruments used, and the time points when they were collected.

**TABLE 1:** Measures at T1 (2010), T2 (2012), and T3 (2017).

Measure	ELPI Instrument	Time point
1. Children’s receptive vocabulary	TVIP (Test de Vocabulario en Imágenes Peabody; Peabody Picture Vocabulary Test).	T1 T2 T3
2. Children’s math skills	Woodcock-Muñoz battery subtests: Calculation, math fluency, and applied problems.	T3
3. Children’s language skills	TEPSI (Test de Desarrollo Psicomotor; Test of Psychomotor Development): Language subtest.	T1
4. Children’s executive functions	Head Toes Knees Shoulders (HTKS) and Backward Digit Span Task (BDST).	T2
5. Mothers’ cognitive skills (vocabulary and working memory)	Wechsler Adults Intelligence Scale 3rd version (WAIS-III): Vocabulary and Digit span subtests.	T1
6. Learning materials	Home Observation for Measurement of the Environment (HOME): Learning materials subscale.	T2

### 2.4 Measures

See Table 1 for a summary of the measures used at each time point.

#### 2.4.1 Children’s receptive vocabulary

To measure receptive vocabulary, we used TVIP (Test de Vocabulario en Imágenes Peabody), the Hispanic adaptation of the Peabody Picture Vocabulary Test (PPVT), validated with the population of Mexico and Puerto Rico (Dunn et al., 1986). It is individually administered to children aged 30 months and older, evaluating the scope of vocabulary acquisition and children’s verbal ability or intelligence.

The evaluator says a word and children choose the image that represents the word by pointing to one of four images in a booklet. TVIP has 125 items in order of increasing difficulty and uses words that are common in Spanish. It consists of a practice phase and an evaluation phase. The practice phase does not add to the raw score. Correct items are scored with 1 and incorrect items are scored with 0. The raw score is obtained by subtracting the number of mistakes from the last item applied. Standard TVIP scores are obtained using Hispanic norms.

The split-half reliability of the test is 0.93 (mean correlation corrected by the Spearman-Brown formula). TVIP was adapted for the Chilean population presenting very high internal consistency (*K-R* = 0.98) and a correlation of.95 between both versions (Strasser et al., 2010).

#### 2.4.2 Children’s math skills

We used three subtests of the Woodcock-Muñoz battery (calculation, math fluency, and applied problems), the Spanish version of the Woodcock-Johnson Battery (Woodcock et al., 2005). Calculation contains 45 items that increase in difficulty, and it measures the ability to perform mathematical calculations (addition, subtraction, multiplication and division operations, and combinations of the four). Math fluency consists of 160 items, and it assesses the ability to quickly solve simple addition, subtraction, and multiplication within three minutes. Applied problems include 62 items increasing in difficulty, and it measures the ability to analyze and solve mathematical problems. The child needs to listen to the formulation of the problem, recognize the procedures to follow, and perform calculations. Visual support material is presented. For all subtests, the score is 1 for correct items and 0 for incorrect items.

The internal consistency reliability coefficients of these subscales are approximately 0.90. The reliability coefficients and standard errors of measurement from The Woodcock-Muñoz battery approximate those obtained from the Woodcock-Johnson III norming sample (Woodcock et al., 2005).

#### 2.4.3 Children’s language skills

We used the Language subtest of the TEPSI (Test de Desarrollo Psicomotor, Test of Psychomotor Development; Haeussler and Marchant, 2003). TEPSI is a screening instrument designed and standardized in Chile for children between 2 and 5 years of age.

The Language subtest (24 items) evaluates receptive and expressive language through tasks such as naming objects, defining words, and verbalizing actions or describing scenes represented in sheets. If the item task is approved, 1 point is awarded; if it is not, 0 point is awarded. All items are applied. The raw score is calculated by adding the scores. The subtest should not be discontinued despite failure on successive items. Internal consistency (*K-R* = 0.94) and interrater reliability (*r* = 0.97) were high. The language subscales from the Stanford Binet and TEPSI were highly correlated (*r* = 0.73).

#### 2.4.4 Children’s executive functions

We used the Head Toes Knees Shoulders (HTKS; Ponitz et al., 2009; Ponitz et al., 2008) and the Backward Digit Span Task (BDST; Davis and Pratt, 1995) to assess children’s executive functions. Both were administered in Spanish.

The HTKS integrates three executive function components: inhibitory control (the child must inhibit the dominant response of imitating the examiner), working memory (the child must remember the rules of the task), and attention focusing (the child must focus on the directions presented by the examiner).

HTKS is a structured observational task, a game-like measure appropriate for children aged 4–8 years. First, the child is asked to follow the rules (touch your head/toes). Then, Part 1 requires one rule pairing (head and toes) and responding opposite of what the examiner says (e.g., touching their head when the examiner says “touch your toes”). If children pass the head/toes part of the task, they move on to Part 2, where the knees and shoulders commands are added. If children respond correctly, they move on to Part 3, which switches Part 2 rules (head goes with knees and shoulders go with toes). Items receive scores of 0 when incorrect, 1 when children self-correct (any motion to the incorrect response, but self-correcting and ending with the correct action), and 2 when correct. The task takes approximately 5–7 min and has strong inter-rater reliability (κ = 0.90).

BDST measures working memory, the child’s ability to hold an instruction in their short-term memory and operate with it. The child is asked to repeat in reverse order a series of numbers that the examiner says aloud at the rate of one number per second. The practice trial consists of 3 series of 2-digit length, with a maximum of four trials with feedback. The evaluation phase consists of 4 levels of difficulty (2-, 3-, 4,-, and 5-digit length, respectively) with 3 series each. The evaluation phase begins with the first level (3 series of 2-digit length) and stops when the child reaches the ceiling (the highest level at which the child is no longer able to answer successfully, when 3 consecutive failures occur). Correct answers are scored 1 and incorrect answers are scored 0. The raw score is the greatest length of digits (2, 3, 4, or 5) where the child had at least one success.

BDST is based on the Digit Retention subtest of the Wechsler Intelligence Scale for Children (WISC-IV; Wechsler, 2003). Cronbach’s alpha of 0.72 has been reported for BDST in a Chilean sample (Guzmán et al., 2019).

#### 2.4.5 Mothers’ cognitive skills (vocabulary and working memory)

We used the Vocabulary and Digit Span subscales of the Wechsler Adults Intelligence Scale 3rd version (WAIS-III; Wechsler, 1954) to measure mothers’ cognitive skills (vocabulary and working memory skills, respectively). WAIS-III was adapted for Chilean population with Cronbach’s alpha over 0.90 for most subtests (Apfelbeck and Hermosilla, 2000).

##### 2.4.5.1 Vocabulary subscale

The evaluator asks the meaning of a set of words. Evaluators start with item 4. If the person gives answers of at least one point to items 4–8, then items 1–3 are considered correct. However, if the person gives a response of 0 points to any of items 4–8, then items 1–3 should be administered and scored. Suspension criterion is 5 consecutive items of 0 points answers. The score is 0 or 2 points for items 1–3, and 0, 1 or 2 for items 4–40. Once the application is finished, all points are added. The maximum raw score possible is 80 points (40 items with the maximum score of 2 points).

##### 2.4.5.2 Digit span subscale

The evaluator asks the person to repeat strings of digits with different lengths and order (forward and backwards). Digit span is measured as the longest string of digits a person can repeat without mistakes. This subscale assesses the ability to manipulate information in working memory.

Digit span forward (ascending order) and digit span backwards (reverse order) are consecutively administered. In digit span forward, the score equals the number of digits repeated without error in the longest series. The maximum raw score is 9 points. In digit span backwards, the score equals the number of digits repeated in reverse order without error in the longest series. The maximum raw score is 8 points. Additionally, a categorical score is provided classifying the assessed skills as: very superior, superior, high average, average, low average, borderline, or extremely low. Scores ranging from average to very superior would indicate positive performance of working memory.

#### 2.4.6 Learning materials

We used the Learning materials subscale of the Home Observation for Measurement of the Environment (HOME; Caldwell and Bradley, 1984). HOME measures the quality and quantity of stimulation and support available to the child in the home environment. Information is obtained through observation and interview with the primary caregiver. The child is physically present and active during the interview.

In 2012 (T2), two versions of HOME were applied: HOME 1 for children up to 3 years old and HOME 2 for 3–7-year-olds. For this study, we used the Learning materials subscale of HOME 2, which consists of seven items assessing the presence and number of games, puzzles, toys, and books available at home that can be used to stimulate learning. Items are answered by the evaluator once the home visit is completed and are scored as Yes (1) or No (0). The raw score is obtained by adding the score of all items. Cronbach’s alpha of 0.74 has been previously reported with a Chilean sample (Bustos Correa et al., 2001).

#### 2.4.7 Maternal capital

We created this latent variable by loading mothers’ educational level, vocabulary skills, working memory, and family income. Maternal capital is based on the concept of human capital (investing in human resources that can influence future outcomes; Becker, 1962) and the example of Lombardi and Dearing (2021), who created a latent variable called “maternal human capital characteristics,” consisting of mothers’ educational level and vocabulary skills.

### 2.5 Data analysis

We used raw scores for all analyses. We conducted descriptive analysis, Spearman’s correlations, and Structural Equation Modeling (SEM), using the lavaan library (version 0.6–16; Rosseel, 2012), with Maximum Likelihood (ML) estimation. Additionally, we calculated measurement invariance. We ran all analyses in R version 4.0.4 (2021-02-15) (R Core Team, 2021).

## 3 Results

### 3.1 Descriptive statistics

Table 2 shows descriptive statistics for children’s scores at ages 3 (T1), 5 (T2), and 11 (T3) (receptive vocabulary, math calculation, fluency and applied problems, language skills, EFs), mothers’ cognitive skills scores at T1 (vocabulary and WM), and home learning materials at T2.

**TABLE 2:** Descriptive statistics of main measures.

Source	Variables	Time point	*N*	*M*	*SD*	Range
Child	Receptive vocabulary	T1	3,739	18.67	14.09	0–79
		T2	3,745	45.99	17.62	0–101
		T3	3,719	96.01	15.59	0–123
	Math calculation	T3	3,733	15.53	4.22	0–36
	Math fluency	T3	3,735	45.12	16.19	0–157
	Math applied problems	T3	3,737	34.89	5.64	0–62
	Language (TEPSI)	T1	3,744	14.91	6.6	0–24
	EF (HTKS)	T2	3,743	23.88	14.06	0–40
	EF (BDST)	T2	3,716	0.74	0.87	0–4
Mother	WM skills	T1	3,754	8.77	1.98	0–17
	Vocabulary skills	T1	3,754	32.82	17.15	0–78
Home	Learning materials	T2	3,731	3.34	2.34	0–7

EFs, Executive Functions; HTKS, Head Toes Knees Shoulders; BDST, Backward Digit Span Task; WM, Working Memory.

### 3.2 Zero-order correlations

We examined the strength of the association between measures (see Table 3). We found moderate to strong correlations between family income and maternal education at T1 (*r* = 0.38), mothers’ WM and vocabulary (*r* = 0.40), children’s language and receptive vocabulary at T1 (*r* = 0.76), children’s EFs measures at T2 (*r* = 0.47), children’s math skills at T3 (0.54 ≤ *r* ≤ 0.60), providing further support for creating our latent variables next.

**TABLE 3:** Zero-order correlations.

Variables	1	2	3	4	5	6	7	8	9	10	11	12	13	14	15
1. Income T1	1	1	1	1	1	1	1	1	1	1	1	1	1	1	1
2. Maternal educational level T1	0.38														
3. Maternal WM T1	0.19	0.26													
4. Maternal vocabulary T1	0.27	0.38	**0.40**												
5. Learning materials T2	0.20	0.23	0.13	0.23											
6. Gender	0.03ns	0.02ns	0.02ns	0.02ns	−0.02ns										
7. Language T1	0.14	0.18	0.14	0.21	0.14	−0.06									
8. Receptive vocabulary T1	0.16	0.18	0.16	0.24	0.14	−0.02ns	**0.76**								
9. EF HTKS T2	0.11	0.12	0.09	0.13	0.13	−0.04**	**0.45**	0.39							
10. EF BDST T2	0.10	0.15	0.12	0.16	0.13	−0.03*	**0.50**	**0.47**	**0.47**						
11. Receptive vocabulary T2	0.15	0.17	0.11	0.19	0.17	−0.04	**0.58**	**0.58**	**0.49**	**0.49**					
12. Receptive vocabulary T3	0.16	0.22	0.14	0.26	0.18	0.05**	0.36	**0.40**	0.30	0.34	**0.45**				
13. Math applied problems T3	0.16	0.19	0.13	0.20	0.14	0.04*	0.34	0.31	0.30	0.37	0.35	**0.44**			
14. Math fluency T3	0.10	0.14	0.08	0.12	0.12	0.08	0.28	0.25	0.26	0.31	0.27	0.29	**0.54**		
15. Math calculation T3	0.15	0.18	0.14	0.18	0.13	−0.01ns	0.27	0.25	0.25	0.30	0.27	0.35	**0.60**	**0.60**	

Only not significant (ns), *p* < 0.05 (*), and *p* < 0.01 (**) are marked. Unmarked coefficients are *p* < 0.001. Effect sizes >0.40 are in bold.

We also found correlational evidence showing that mothers’ educational level, vocabulary skills, working memory, and family income measured at T1 had a positive relation with children’s math and receptive vocabulary at T3 (0.10 ≤ *r* ≤ 0.26).

Additionally, we found a small negative correlation between children’s gender and language at T1 and EFs measures and receptive vocabulary at T2 (favoring girls), and a small positive correlation between children’s gender and receptive vocabulary and math applied problems and fluency at T3 (favoring boys) (0.03 ≤ | *r*| ≤ 0.08). Therefore, girls had a slight early advantage in language and EFs, but later boys had a slight advantage in vocabulary and math.

### 3.3 Structural equation modeling

To address our main research question—do home learning materials, children’s EFs and receptive vocabulary at age 5 (T2) mediate the relation between mother’s SES and cognitive skills and children’s language at age 3 (T1) with children’s math and receptive vocabulary at age 11 (T3)?—we used structural equation modeling (SEM).

To test our proposed model, we created five latent variables: SES, maternal cognitive skills, and children’s language at T1, children’s EFs at T2, and children’s math at T3. To test model fit, we used a model test statistic and four approximate fit indexes: Model chi-square (χ^2^) with its degrees of freedom, Comparative Fit Index (CFI), Non-Normed Fit Index (NNFI), Standardized Root Mean Square Residual (SRMR), and Root Mean Square Error of Approximation (RMSEA). Fit index values indicating good model fit are > 0.95 for CFI and NNFI, < 0.08 for SRMR, and < 0.05 for RMSEA (Kline, 2016).

**FIGURE 1:**
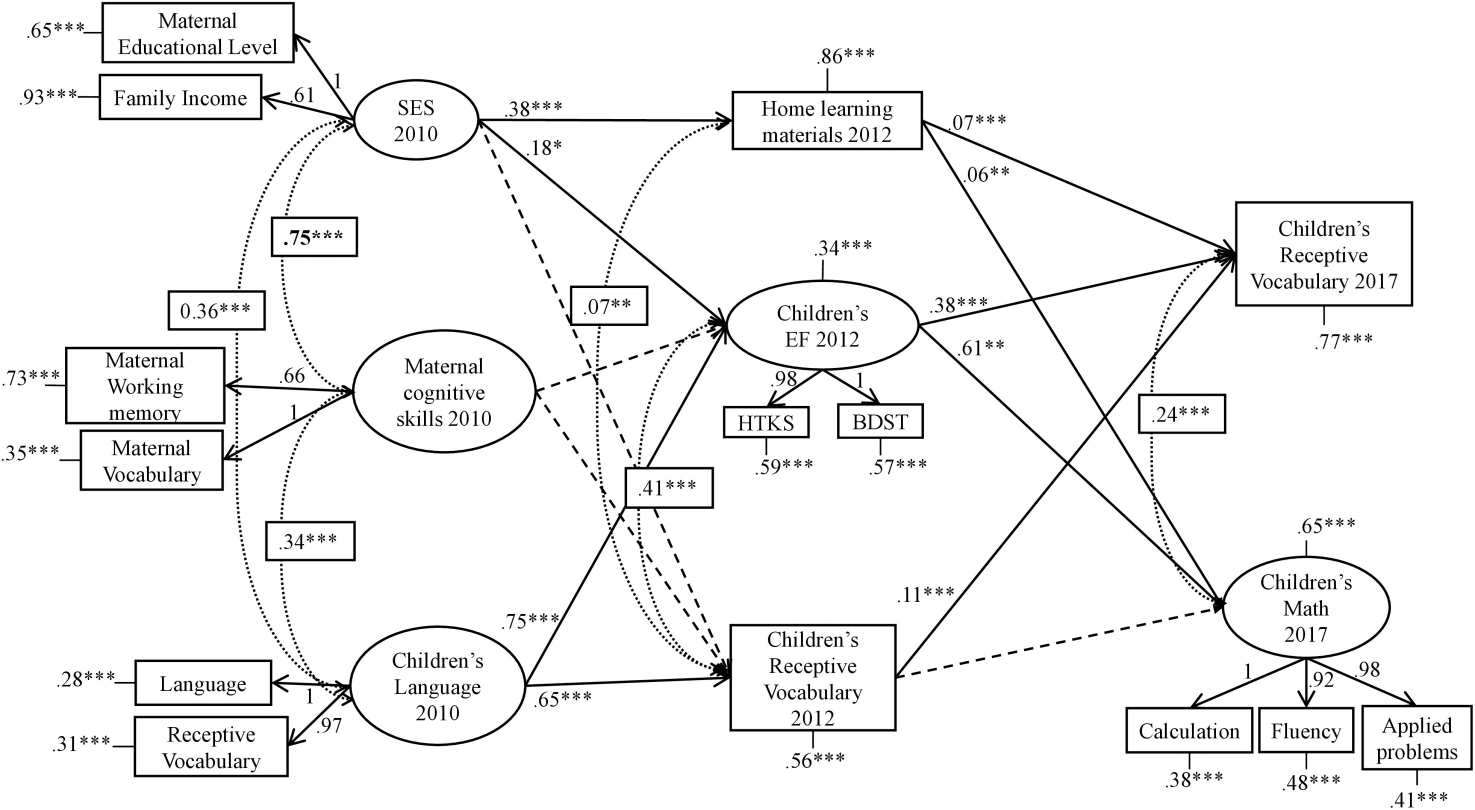
Graphical representation of the initial model tested in SEM. The figure indicates data collection years: 2010 (T1), 2012 (T2), and 2017 (T3). Only significant standardized estimates are shown. Dashed straight lines represent non-significant paths. Covariances estimates are presented in squares. Observed variables are represented with rectangles and latent variables with ellipses. Collinearity presented in bold. *p < 0.05, **p < 0.01, ***p < 0.001.

Our initial structural model test results are displayed in Figure 1 [χ^2^(60) = 306.066, *RMSEA* = 0.038 [90% CI (0.034–0.042)], *SRMR* = 0.026, *CFI* = 0.978, *NNFI* = 0.967]. This model did not fit the data well, as we detected a multicollinearity problem between two independent variables (SES and maternal cognitive skills), which were highly correlated (0.75) (Supplementary Table 3). Therefore, the estimates of these predictors were unreliable (Cohen et al., 2003). To address this problem, we specified this model creating a latent variable called “maternal capital,” which consisted of mothers’ educational level, vocabulary skills, working memory, and family income (please see Methods for details).

**FIGURE 2:**
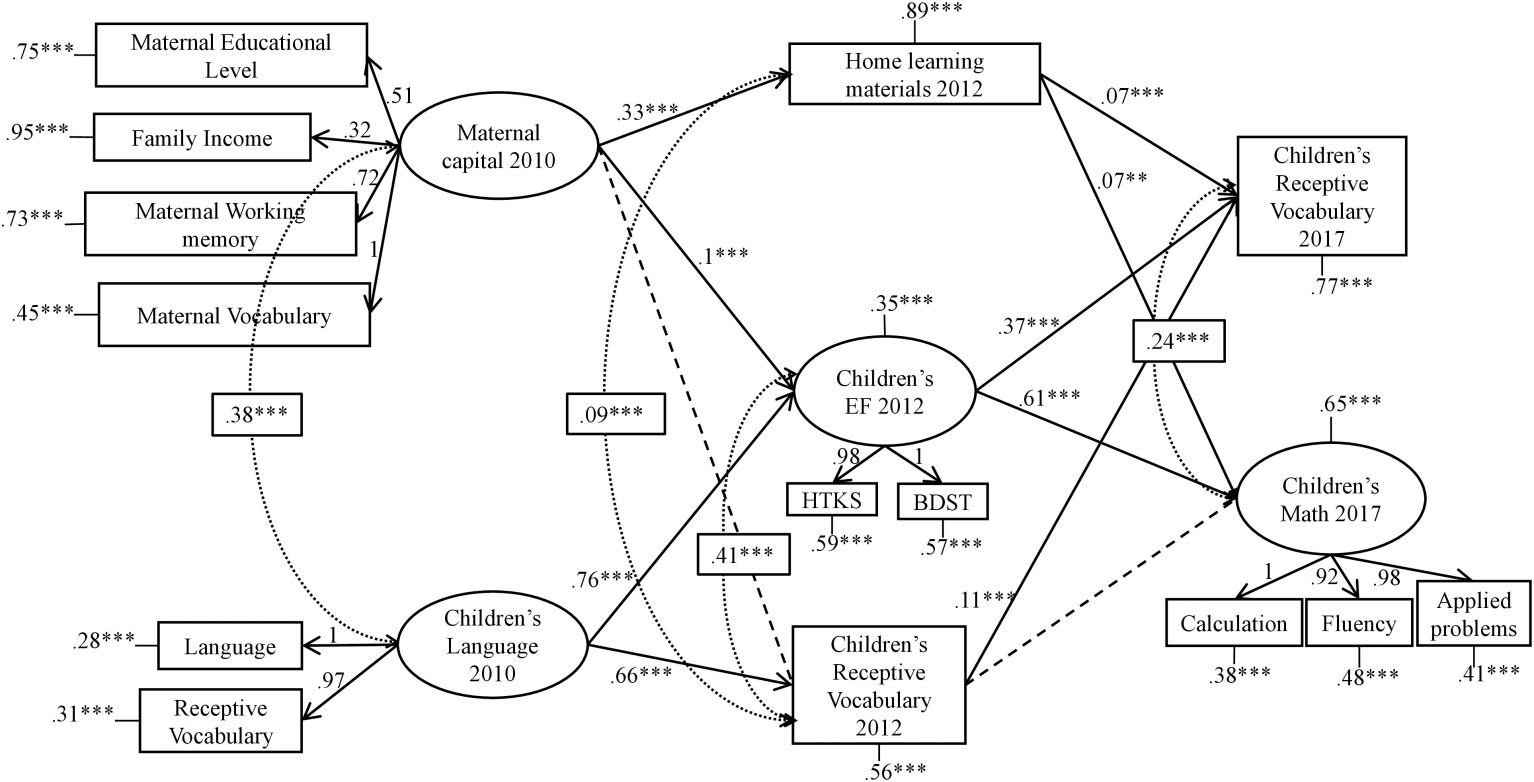
Graphical representation of the final model tested in SEM. Only significant standardized estimates are shown. Dashed straight lines represent non-significant paths. Covariances estimates are presented in squares. Observed variables are represented with rectangles and latent variables with ellipses. *p < 0.05, **p < 0.01, ***p < 0.001.

Our refined structural model (shown in Figure 2 and Table 4) fitted the data well [χ^2^ (64) = 358.459, *RMSEA* = 0.040 [90% CI (0.036–0.044)], *SRMR* = 0.030, *CFI* = 0.974, *NNFI* = 0.963], with no collinearity issues (Table 5). Hence, we accepted this as our final model.

**TABLE 4:** Final model: path summary.

Path	β	SE	*p*	95% CI
Children’s Math 2017 ∼ Home learning materials 2012	0.06	0.02	0.002	(0.02, 0.10)
Children’s math 2017 ∼ Children’s EFs 2012	0.61	0.04	0.000	(0.54, 0.68)
Children’s Math 2017 ∼ Children’s receptive vocabulary 2012	−0.05	0.04	0.155	(-0.12, 0.02)
Children’s receptive vocabulary 2017 ∼ Home learning materials 2012	0.07	0.02	0.000	(0.03, 0.10)
Children’s receptive vocabulary 2017 ∼ Children’s EFs 2012	0.37	0.03	0.000	(0.31, 0.44)
Children’s receptive vocabulary 2017 ∼ Children’s receptive vocabulary 2012	0.11	0.03	0.000	(0.05, 0.17)
Home learning materials 2012 ∼ Maternal capital 2010	0.33	0.02	0.000	(0.29, 0.37)
Children’s EFs 2012 ∼ Maternal capital 2010	0.10	0.03	0.000	(0.05, 0.15)
Children’s EFs 2012 ∼ Children’s language 2010	0.76	0.02	0.000	(0.73, 0.80)
Children’s receptive vocabulary 2012 ∼ Maternal capital 2010	0.01	0.02	0.558	(-0.03, 0.05)
Children’s receptive vocabulary 2012 ∼ Children’s language 2010	0.66	0.02	0.000	(0.63, 0.69)

β, Standardized estimates; SE, Standard Error; CI, 95% confidence intervals; p, *p*-values.

**TABLE 5:** Covariances in final model tested in SEM.

Covariance	est.std	SE	*p*	95% CI
Children’s EFs 2012 ∼∼ Home learning materials 2012	0.07	0.03	0.059	(0.00–0.13)
Home learning materials 2012 ∼∼ Children’s Receptive Vocabulary 2012	0.09	0.02	0.000	(0.05–0.13)
Children’s EFs 2012 ∼∼ Children’s receptive vocabulary 2012	0.41	0.03	0.000	(0.35–0.48)
Maternal capital 2010 ∼∼ Children’s language 2010	0.38	0.02	0.000	(0.33–0.42)
Children’s math 2017 ∼∼ Children’s receptive vocabulary 2017	0.24	0.02	0.000	(0.19–0.28)

est.std, Standardized estimates; SE, Standard Error; CI, 95% confidence intervals; p, *p*-values.

Findings from the structural model indicated that Maternal capital at T1 correlated with home learning materials and children’s EFs at T2; children’s language at T1 correlated with children’s EFs and receptive vocabulary at T2; home learning materials and children’s EFs at T2 correlated with children’s receptive vocabulary and math at T3; finally, children’s receptive vocabulary at T2 correlated with children’s receptive vocabulary at T3. On the other hand, we found two non-significant paths: Maternal capital at T1 and children’s receptive vocabulary at T2, and children’s receptive vocabulary at T2 and children’s math at T3. Therefore, our hypotheses were partially corroborated.

The strongest indirect paths started with children’s language at T1 correlating with children’s EFs at T2, which in turn correlated with children’s math [est.std = 0.47 [95% CI (0.40–0.53)], SE = 0.03, *p* = 0.000] and receptive vocabulary at T3 [est.std = 0.29 [95% CI (0.23–0.34)], SE = 0.03, *p* = 0.000]. Notably, three out of four indirect paths with receptive vocabulary at T2 as a mediator were non-significant, whereas the paths with home learning materials at T2 as a mediator were weak, and paths starting with maternal capital at T1 were either non-significant or weak. Therefore, EFs at T2 proved to be the best mediator between children’s language at T1 and both math and vocabulary skills at T3 (Table 6).

**TABLE 6:** Final model: indirect effects.

Indirect paths	est.std	SE	*p*	95% CI
Maternal capital 2010 → Home learning materials 2012 → Children’s math 2017	0.02	0.01	0.002	(0.01–0.03)
Maternal capital 2010 → Children’s EFs 2012 → Children’s math 2017	0.06	0.02	0.000	(0.03–0.09)
Maternal capital 2010 → Children’s receptive vocabulary 2012 → Children’s math 2017	0.00	0.00	0.588	(0.00–0.00)
Children’s language 2010 → Children’s EFs 2012 → Children’s math 2017	0.47	0.03	0.000	(0.40–0.53)
Children’s language 2010 → Children’s receptive vocabulary 2012 → Children’s math 2017	−0.03	0.02	0.156	(-0.08–0.01)
Maternal capital 2010 → Home learning materials 2012 → Children’s receptive vocabulary 2017	0.02	0.01	0.000	(0.01–0.03)
Maternal capital 2010 → Children’s EFs 2012 → Children’s receptive vocabulary 2017	0.04	0.01	0.000	(0.02–0.06)
Maternal capital 2010 → Children’s receptive vocabulary 2012 → Children’s receptive vocabulary 2017	0.00	0.00	0.563	(0.00–0.01)
Children’s language 2010 → Children’s EFs 2012 → Children’s receptive vocabulary 2017	0.29	0.03	0.000	(0.23–0.34)
Children’s language 2010 → Children’s receptive vocabulary 2012 → Children’s receptive vocabulary 2017	0.08	0.02	0.000	(0.04–0.11)

est.std, Standardized estimates; SE, Standard Error; CI, 95% confidence intervals; p, *p*-values.

### 3.4 Measurement invariance

Additionally, we investigated whether the final model was valid for girls and boys (i.e., if the model interpretation would be the same for girls and boys). We calculated measurement invariance grouping by gender, to test model sensitivity to group differences. For this purpose, we estimated and compared the model fit indices of increasingly constrained models: Configural, Metric, Scalar. We observed that fit indices did not meet conventional standards when grouping by gender (Kline, 2016).

However, a chi-squared difference test comparing the Configural and Metric invariance models was not significant (*p* = 0.3931), which meant that Metric invariance was established, and we could test Scalar invariance. A chi-squared difference test comparing the Metric and Scalar invariance models was significant (*p* < 0.001), which might suggest lack of Scalar invariance. However, the CFI change between the Metric and Scalar models was 0.003 in absolute value, indicating that constraints did not worsen model fit (see Table 7). As this effect size was below the cutoff point of 0.1, the assumption of Scalar invariance could be maintained, which means that the model fits equivalently across groups (Hirschfeld and von Brachel, 2014). Although the final SEM model showed good model fit, model fit declined when grouping by gender. Despite this, the invariance testing indicated that the model functioned equivalently for girls and boys.

**TABLE 7:** Model fit indices for models with configural, metric, scalar invariance with gender as a grouping variable.

Model	χ ^2^	df	RMSEA	SRMR	TLI	CFI	AIC	BIC
Configural	2175.190	156	0.096	0.115	0.792	0.846	115,690	116,367
Metric	2182.543	163	0.094	0.115	0.801	0.846	115,683	116,319
Scalar (partial)	2236.145	174	0.092	0.115	0.810	0.842	115,715	116,285
Metric-configural	7.353	7	−0.002	0.000	0.009	0.000	−6.647	−48.266
Scalar-Metric	53.603	11	−0.002	0.000	0.009	−0.003	31.603	−33.799

## 4 Discussion

The aim of this study was to test whether home learning materials, children’s EFs and receptive vocabulary at age 5 (T2) mediate the relation between mother’s SES and cognitive skills and children’s language at age 3 (T1) with children’s math and receptive vocabulary at age 11 (T3). We contribute to the literature by revealing indirect longitudinal paths promoting children’s math and vocabulary. Although previous studies have tested predictive models of children’s math and vocabulary, many of these analyzed decades-old data, mostly from Western countries, were non-representative of their population, included few measures, and only some covered different stages of development (Burchinal et al., 2020; Lombardi and Dearing, 2021; Wade et al., 2018; Waters et al., 2021). The present study adds to the literature by testing a comprehensive model, following children across infancy, preschool, and primary school, including novel predictors (such as mothers’ cognitive skills) and three math skills (calculation, fluency, and applied problems). We used SEM to test this model on a sample of 3,757 children from Chile, enrolled in ELPI across three waves (years 2010, 2012, 2017). The large sample size and robust methodological approaches strengthen the credibility of our results.

Indirect effects showed that children’s EFs in preschool were the strongest mechanism through which early language skills influence math and receptive vocabulary in primary school. Contrary to our expectations, three out of four indirect paths testing receptive vocabulary as a mediator were non-significant. Besides, paths testing home learning materials as a mediator had a weak effect. Finally, indirect paths that started from maternal capital at T1 were either weak or non-significant. These findings provide an important addition to our understanding of developmental processes.

The strongest indirect path began with early language skills, which were correlated to children’s EFs during preschool. It has been suggested that gains in language can facilitate children’s more complex thinking and the use of linguistic strategies, which in turn may support the development of EFs skills (Xing et al., 2022). In addition, children’s EFs had the strongest effect on both math and receptive vocabulary in primary school. Thus, the development of children’s math and receptive vocabulary appeared to rely on their EFs measured 5 years earlier.

While some studies have focused solely on one EFs skill, such as inhibitory control (Wade et al., 2018), the present study included three EFs skills: WM, inhibitory control, and attention focusing. These are necessary skills to be able to carry out developmental tasks that demand focus, holding the instruction in mind, selecting the correct answer while inhibiting incorrect ones (Diamond, 2013). Previous studies have shown that EFs are important in the classroom learning context, for example by facilitating problem-solving, staying focused in class, and persisting on a challenging activity, thereby supporting academic achievement (Burchinal et al., 2020; Sektnan et al., 2010; Xing et al., 2022).

Although EFs were beneficial for both math and receptive vocabulary skills, the effect of the indirect path was stronger for math than receptive vocabulary (0.47 vs. 0.29). This difference may suggest that EFs more strongly support math calculation, fluency, and solving applied problems than tasks involving the recognition of concepts from a set of alternatives.

Early language skills had the strongest effect on both EFs and receptive vocabulary in preschool. Hence, early language development was an important foundation for children’s development of EFs and receptive vocabulary 2 years later. These results are consistent with previous international studies (Burchinal et al., 2020; Xing et al., 2022) and prior studies analyzing receptive vocabulary at T1 and T2 using ELPI (Berthelon et al., 2020).

On the other hand, maternal capital correlated with children’s receptive vocabulary and math in primary school through home learning materials and children’s EFs in preschool. Surprisingly, maternal capital did not correlate with children’s vocabulary in preschool. Regarding the correlation between maternal capital and home learning materials and children’s EFs in preschool, families with higher SES have the financial resources to invest in learning materials, whereas higher education influences parents’ beliefs and behaviors regarding their children’s cognitive development (Sektnan et al., 2010; Waters et al., 2021).

Unexpectedly, even though maternal capital and language skills were strongly related at T1, maternal capital did not have a significant effect on receptive vocabulary 2 years later. Previous Chilean studies using ELPI have found a longitudinal relation between maternal education and receptive vocabulary and between maternal vocabulary and receptive vocabulary (Berthelon et al., 2020). We could hypothesize that our inclusion of family income and maternal WM skills may have influenced the relation between maternal capital and receptive vocabulary, as these family variables may not be as relevant for receptive vocabulary 2 years later as early language skills were.

Home learning materials correlated with math and receptive vocabulary in primary school. This positive relation has been found in previous international research (Zadeh et al., 2010) and is in line with a previous study of learning materials and receptive vocabulary using ELPI (Abufhele et al., 2022). As children enter school, this new context might become more influential for the development of math and vocabulary than earlier home experiences (Melhuish et al., 2019), which could explain the weaker 5-year effect of learning materials in comparison to EFs. Another explanation for this weak effect might be related to the conceptualization of learning materials. Some studies have considered learning materials as part of a wider home environment measure (Lehrl et al., 2016), measured specific materials (e.g., children’s books or numeracy resources; Shahaeian et al., 2018), or used the HOME learning materials subscale as we did (Zadeh et al., 2010). Even though Zadeh et al. (2010) used the HOME learning materials subscale, they carried out a cross-sectional study with first grade children, which might also help understand why we found a weaker effect, as we analyzed a 5-year longitudinal effect.

Receptive vocabulary in preschool correlated with receptive vocabulary in primary school. A previous study also found a positive relation between preschool and primary school receptive vocabulary (Burchinal et al., 2020). However, we did not replicate their longitudinal relation between preschool receptive vocabulary and primary school math. One difference between our studies is our inclusion of three math tasks (fluency, calculation, and applied problems), whereas Burchinal et al. (2020) only used applied problems, a task that requires children to analyze and solve word problems and therefore more likely to rely on vocabulary skills.

As the main indirect path predicting math and receptive vocabulary in primary school included early language skills and EFs in preschool, these skills stand out as potential targets for intervention. Children between 2.5 and 4.5 years of age (T1) may have different caregiving experiences, as they may be looked after at home or nursery, or other kinds of arrangements. In early childhood, it is important for children to be exposed to a variety of words and have opportunities to engage in talks with more advanced speakers (Wade et al., 2018). Caregivers can support their children by naming objects in their environment, defining new words, and helping them verbalize actions.

Furthermore, children’s EFs were assessed when they were between 4.5 and 7 years old (T2). This period encompasses preschool to first grade, when children have their first experiences with the school system. Educators may use this window of time as an opportunity to promote EFs (WM, inhibitory control, and attention focusing) in the classroom and make recommendations for parents on activities they might be able to do at home. Policymakers should consider the wide evidence supporting the key role that EFs plays for school readiness (Diamond, 2013). Our study contributes further evidence that EFs in the preschool years have a strong effect on school-relevant skills even 5 years later, when children in our sample were in fourth to sixth grades. Therefore, it would be advantageous to promote gains in WM, inhibitory control, and attention focusing in the preschool classroom, e.g., through group and individual activities increasing in difficulty over time as suggested by Röthlisberger et al. (2012). Addressing these early targets—early language skills and EFs in preschool—can set the foundation for children to develop their receptive vocabulary and math skills when they are between 9.5 and 12 years of age.

Our model applied for both girls and boys; however, future studies could further analyze why fit indices worsened when grouping by gender. One explanation may be related to the small gender differences we noted: girls having a slight advantage in early language and EFs and boys having a slight advantage in later vocabulary and math. Our results further inform previous evidence-based suggestions to improve early childhood education in Chile: a study with pre-K classrooms found that three domains of teacher-child interaction (classroom organization, emotional and instructional support) were related with children’s vocabulary, early writing and numeracy, and EFs skills (Leyva et al., 2015). Also, improving the quality of classroom practices and children’s preschool attendance have been suggested as targets to support children’s learning in this population (Yoshikawa et al., 2015).

Our findings point to the importance of early experiences for the development of math and vocabulary during the primary school years. These findings are timely and relevant, showing the complexity of children’s development of receptive vocabulary and math skills. This evidence can be useful for parents, teachers, and policy makers to support children.

### 4.1 Limitations and future studies

There are a range of limitations that need to be acknowledged: fathers’ cognitive skills (vocabulary and working memory) were not assessed on ELPI at T1. Therefore, we suggest that future longitudinal studies include fathers at each test point, as their characteristics play a significant role in their children’s developmental trajectories (Garon-Carrier et al., 2018). Secondly, math skills were only measured at T3 when children were at school. We would recommend that math skills are considered from preschool, as early math skills have been related to EFs and they predict math achievement in school (Lehrl et al., 2016). Besides, reading was not measured at T3, therefore we included receptive vocabulary as a literacy outcome. It would be valuable to include this outcome in future waves.

Another limitation regards the gaps between ELPI waves (2010, 2012, 2017). Future childhood longitudinal studies may want to consider a smaller age range but more continuous waves of measurement. Doing so would allow to further analyze the underlying mechanisms of the relationships that have been found with the available data. Furthermore, although differences between our sample and the sample with no follow-up data were not significant at a threshold of *p* < 0.001 (suggesting that attrition did not lead to significant biases) we acknowledge that attrition may potentially limit the generalizability of results. Despite our strong methodological approach, there is risk of potential omitted variable bias, such as school-level factors (quality of early childhood education or teacher-child interactions). Finally, although these are longitudinal relations, we cannot establish causality.

Future studies could follow up on how our model evolves once children enter high school. Another interesting addition would be if and how parents engage in the use of learning materials with their children to promote math and vocabulary development (e.g., what kind of activities they do, how do they keep their child’s attention, what strategies they use; Wei et al., 2019). More detailed information on the effect of home practices on children’s outcomes can guide parents and inform the design of interventions. Even though there are limitations associated with carrying out secondary analyses (e.g., not being part of the decision-making process), we believe that the benefits (e.g., large sample size, inclusion of families across the country, three waves of measurement over 7 years, etc.) outweigh these limitations and results provide valuable insights of children growing up in Chile.

### 4.2 Conclusion

We tested a 7-year longitudinal model over three time points at different stages of development (infancy, preschool, and primary school). EFs in preschool were the strongest mediator linking children’s language in infancy with math and vocabulary skills in primary school. To our knowledge, this is the first report on a nationally representative sample followed for 7 years and testing a mediation longitudinal model predicting children’s math and receptive vocabulary. We contribute information from Latin America, an underrepresented region in research with high levels of educational inequity. We found two children’s skills that are worth targeting to promote children’s math and receptive vocabulary in primary school: language skills in infancy and EFs during the preschool years. This study has the potential to make a significant contribution to developmental science. The rigorous methodology, the longitudinal design, and the novel findings offer meaningful insights to our understanding of long-term developmental processes in childhood.

## Data Availability

Publicly available datasets were analyzed in this study. This data can be found at: https://observatorio.ministeriodesarrollosocial.gob.cl/elpi-tercera-ronda.
