# Supplementary material for: A 7-year longitudinal model of math and receptive vocabulary in primary school
Source: Front Psychol. 2026 May 1;17:1689488. doi: 10.3389/fpsyg.2026.1689488 (PMC13175839; doi:10.3389/fpsyg.2026.1689488)
Supplement: Supplementary file 1 [file Table_1.docx]

**Table 1S**

*T-test results comparing missing and study samples*

| T1 Variables | Missing sample  (N = 3,578)  M (SD) | Study sample  (N = 3,757)  M (SD) | *t* | *p* |
| --- | --- | --- | --- | --- |
| Family income | 550,457 (1,357,869) | 458,354 (898,325) | 2.968 | 0.003 |
| Maternal working memory | 7.02 (2.83) | 6.84 (2.70) | 2.777 | 0.006 |
| Maternal vocabulary | 8.23 (3.71) | 8.02 (3.60) | 2.484 | 0.013 |
| Children’s language | 51.37 (11.76) | 51.19 (11.47) | 0.647 | 0.518 |
| Children’s receptive vocabulary | 103.66 (15.53) | 103.85 (15.34) | -0.519 | 0.604 |
| Children’s age | 41.19 (6.90) | 41.05 (6.85) | 0.869 | 0.385 |

*Note.* Missing sample was defined as 30–58-month-old children in 2010 (T1) without follow-up data in the 2012 (T2) and 2017 (T3) waves of the ELPI survey.

**Table 2S**

*Wilcoxon rank sum and Chi-square test results comparing missing and study samples*

| T1 Variables | Missing sample  N | Study sample  N | Coefficient | *p* |
| --- | --- | --- | --- | --- |
| Maternal educational level |  |  | *W =* 6828159 | 0.001 |
| No formal education | 13 | 19 |  |  |
| Primary education | 622 | 727 |  |  |
| High School | 2,424 | 2,550 |  |  |
| Technical studies | 224 | 240 |  |  |
| College degree or higher | 255 | 191 |  |  |
| Gender |  |  | *X^2^_(1)_ =* 0.105 | 0.746 |
| Boys | 1,794 | 1,899 |  |  |
| Girls | 1,784 | 1,858 |  |  |

*Note.* Missing sample was defined as 30–58-month-old children in 2010 (T1) without follow-up data in the 2012 (T2) and 2017 (T3) waves of the ELPI survey.

**Table 3S**

*Covariances in initial model tested in SEM*

| Covariance | est.std | SE | *p* | 95% CI |
| --- | --- | --- | --- | --- |
| Children’s EFs 2012 ~~ Home learning materials 2012 | 0.030 | 0.038 | 0.435 | -0.045 - 0.104 |
| Home learning materials 2012 ~~ Children’s Receptive Vocabulary 2012 | 0.067 | 0.023 | 0.003 | 0.023 - 0.111 |
| Children’s EFs 2012 ~~ Children’s Receptive Vocabulary 2012 | 0.405 | 0.035 | 0.000 | 0.337 - 0.474 |
| SES 2010 ~~ Maternal cognitive skills 2010 | 0.754 | 0.035 | 0.000 | 0.685 - 0.823 |
| SES 2010 ~~ Children’s Language 2010 | 0.364 | 0.030 | 0.000 | 0.304 - 0.424 |
| Maternal cognitive skills 2010 ~~ Children’s Language 2010 | 0.339 | 0.024 | 0.000 | 0.292 - 0.385 |
| Children’s Math 2017 ~~ Children’s Receptive Vocabulary 2017 | 0.236 | 0.023 | 0.000 | 0.190 - 0.281 |

*Note*. Standardized estimates (est.std), Standard Error (SE), 95% confidence intervals (CI), and p-values (p)
